# Unsupervised Classifications of Depression Levels Based on Machine Learning Algorithms Perform Well as Compared to Traditional Norm-Based Classifications

**DOI:** 10.3389/fpsyt.2020.00045

**Published:** 2020-02-14

**Authors:** Zhenkai Yang, Chuansheng Chen, Hanwen Li, Li Yao, Xiaojie Zhao

**Affiliations:** ^1^ School of Artificial Intelligence, Beijing Normal University, Beijing, China; ^2^ Department of Psychological Science, University of California, Irvine, Irvine, CA, United States

**Keywords:** depression, scale data, unsupervised classification, norm, clustering

## Abstract

Large-scale screening for depression has been using norms developed based on a given population at a given time. Researchers have attempted to adjust the cutoff scores over time and for different populations, but such efforts are too few and far in between to be sensitive to temporal and regional variations. In this study, we proposed an unsupervised machine learning approach to constructing depression classifications to overcome the limitations of the traditional norm-based method. Data were collected from 8,063 Chinese middle and high school students. Using k-means clustering, we generated four levels of depressive symptoms to match the norm-based classifications. We then evaluated the validity of the classifications by comparing them with the norm-based method (and its variations) in terms of their robustness, model performance (accuracy, AUC, and sensitivity), and convergent construct validity (i.e., associations with known correlates). The results showed that our automatic classification system performed well as compared to the norm-based method.

## Introduction

Depression is a common, chronic, and recurring condition that imposes a substantial burden on both the afflicted individuals and the society (1). More than 300 million people are now living with depression, which represents an increase of more than 18% between 2005 and 2015 (2). By 2020, depression is expected to become the second most common illness after only cardiovascular diseases. Furthermore, recent years have witnessed a trend towards younger age-of-onset for depression and year-to-year increases in the number of adolescent patients with depression, many of whom also have strong suicidal ideation as well as suicidal behaviors (3, 4). Consequently, it is a public health priority to diagnose and treat depression in both children and adults (5).

However, depression is widely undiagnosed and thus untreated because of various reasons such as societal stigmas about mental disorders, inefficient tools for diagnosis, and inadequate mental-health resources. For example, almost half of the world's population lives in a country with only two psychiatrists per 100,000 people (2), which makes it practically impossible to screen the population for depression with the sanctioned methods of expert interview and clinical diagnosis (6–8). Moreover, such methods are simply too costly and labor-intensive for large-scale screening for depression (9, 10). Consequently, it is out of necessity to use self-report surveys or scales as large-scale screening measures (11, 12). The commonly used scales include Beck Depression Inventory [BDI, (13)], Center for Epidemiologic Studies-Depression [CESD, (14)], and Symptom Checklist 90-Revision [SCL-90-R, (15)]. Such scales have proven to be convenient to administer and to have good psychometric properties such as reliability and validity (16). Researchers have also determined different cutoff points (norms) for various categories of depression. For example, for BDI-13, the score ranges for various levels of depression are 0–4 (No Depression), 5–7 (Mild), 8–15 (Moderate), and 16–39 (Severe). These cutoff points were based on the original study of Beck et al. (13). For CESD, the score ranges are 0–15 (No Depression), 16–19 (Possible Depression), and above 20 (Obvious depression) based on the study by Radloff et al. (14).

Because the original samples used to establish the norms were limited to a given population at a given time, the cutoff points were not assured generalizability to other populations, which might have contributed to the large variations of prevalence rates of depression among different populations (17, 18). It is thus necessary to consider each population's background characteristics (i.e., age, patient status, cultural values, language use, economic development level, etc.) when determining whether the cutoff points would apply (19). Indeed, some researchers modified the cutoff scores or revised the scale for different communities when they used Center for Epidemiologic Studies Depression (CES-D) scale for depression screening (20). They raised the cutoff score of possible depression from 16 to 22 for older people and to 19 for patients with chronic pain. In terms of BDI (full scale), Rui-Fan et al. (21) discussed the lack of consistent cutoff scores in hospitals, with some using 5 as the cutoff score whereas others using 10 or 22 for screening (22–24).

Most relevant to this study, Yang et al. (25) collected data of BDI (full scale) and CES-D from 634 Chinese adolescents. Then they invited 13 graduate students in a clinical psychology program to interview the adolescents using K-SADS (Schedule for Affective Disorder and Schizophrenia for School-age Children) and used DSM-IV (Diagnostic and Statistical Manual of Mental Disorders) as the standard to determine depression diagnosis for these adolescents according to the number of depressive symptoms, duration and degree of functional impairment. Based on the validity criteria of sensitivity, specificity, positive predictive value (PPV), and Area Under the Curve (AUC), Yang et al. proposed adjusted cutoff scores for BDI (full scale) and CES-D for Chinese adolescents. For BDI (full scale), the cutoff score between non-depression and mild depression was adjusted from 14 to 15, while that between moderate depression and severe depression was adjusted from 29 to 28. For CES-D (adolescent), the cutoff score between non-depression and mild depression was adjusted from 20 to 24, while that between moderate depression and severe depression was adjusted from 24 to 29.

Despite the great efforts of previous researchers in adjusting the norms of depression scales established in earlier times and/or a different country, they will likely lag behind the changing times due to the rapid social and technological change. Indeed, there were significant changes in the norms of the SCL-90 in a 13-year period between the years of 1986 and 1999 (26). Yet it has been 21 years since it was renormed. One main reason for the infrequent renorming is the associated costs in money and time. It would be ideal if a data-driven process can be used to classify any given samples into different levels of depression that are sensitive to these samples' characteristics and are reliable and valid.

The current study presents such an attempt using machine learning. As a complete data-driven method, machine learning is an advanced versatile method that can use big data to train and improve models of prediction, classification, and optimization (27). Recently, researchers have used machine learning to predict depression from depression-related factors. For example, Jin et al. (7) used several classic machine learning methods such as logistic regression, multi-layer perception, and support vector machine to predict depression (measured with Patient Health Questionnaire-9 and Patient Health Questionnaire-2) from common demographic factors, health condition, depression history, and other depression-related factors. Victor et al. (28) developed a deep learning model to detect depression (measured with Patient Health Questionnaire-9) based on video questions regarding current mental well-being and demographics data. Sau and Bhakta (29) used random forest to predict depression and anxiety (measured with Hospital Anxiety and Depression Scale, respectively) from socio-demographic variables. All these studies aimed to identify predictors of depression, which was measured with scales such as PHQ and HADS. Their machine learning was based on supervised learning where the samples were all labeled based on their depression scores or levels according to their original cutoff points. These studies are useful in identifying alternative prediction models of depression but do not deal with the issue of the need for sample-specific norms. To accomplish that aim, we need unsupervised machine learning algorithms such as clustering to process unlabeled data. Thus far no study has used such algorithms to construct data-driven classifications of depression.

Several clustering methods are currently used in machine learning and they all have their strengths and weaknesses. We chose k-means clustering for the following reasons. For example, the DBSCAN is a famous density-based clustering method, but unlike k-means clustering, it does not allow us to set a specific number of clusters, which we needed to match the number of depression levels used in the traditional norm-based methods in order for us to compare the results of the two methods. Moreover, the DBSCAN is very sensitive to the set of parameters (radius, threshold) used in modeling. Meanwhile, what we needed was an automatic and stable model with as little manual adjustment of the parameters as possible. K-means clustering meets that requirement. Another advanced clustering method commonly in use now is hierarchical clustering, but it has high computational complexity, which is not suitable for a large amount of data and for easy adoption in real-life application of the method in various situations (schools, hospitals, etc., where there are few data scientists). Other less commonly used clustering methods such as grid-based clustering and model-based clustering also have their own limitations and are thus less ideal than the k-means clustering for the purpose of this study. Taken together, the main advantages of the k-means clustering method included the ability to set a fixed number of theoretically meaningful and practically useful clusters, little need for parameter adjustment, low computational complexity, and easy adoption in various real-life contexts.

In sum, this study used machine learning to develop an automatic classification method to determine levels of depression and assess its performance. Data came from 8,063 middle and high school students from 11 different schools in China. K-means clustering algorithm was used to construct a depression classification based on the data collected with BDI. To assess the reliability and validity of this classification, we compared the classification results with existing norms as well as adjusted norms (see the Methods section) by examining (a) the clustering method's robustness, (b) the correspondence between depression levels based on different methods, (c) the different methods' model performance in cross-validation (accuracy, AUC, and sensitivity), and (d) the associations between depression levels and theoretically related constructs such as stressful life events (measured with Adolescent Self-Rating Life Events Checklist [ASLEC], 27 items) (30), perceived stress (measured with Perceived Stress Scale [PSS], 14 items) (31), anxiety (measured with Self-Rating Anxiety Scale [SAS], 20 items) (32), and sleep quality (measured with Insomnia Severity Index [ISI], 7 items) (33).

## Methods

### Data Collection and Filtering

A total of 8,063 middle and high school students (mean age = 14.4 ± 2.4 years old, range = 10–19) from 11 different schools in China participated in this study. They completed electronic versions of five scales: BDI-13 (13), ASLEC (34), PSS (35), SAS (36), and ISI (37) in their respective schools' computer rooms. This study was approved by the Institutional Review Board of the State Key Laboratory of Cognitive Neuroscience and Learning at Beijing Normal University.

To screen for careless responses, we used the IQR method (38) to filter the data. Data of 368 (4.6%) students were removed, resulting in a final sample of 7,695 students.

### Construction of Depression Classification

K-means clustering was used to classify the levels of depression based on BDI-13, with k set as 4, which was the same as the number of the levels based on the traditional norms. The k-means clustering is conducted in a 13-dimensional space, with the 13 items of BDI being used as the 13 features, i.e., the score of each item as a feature value. At the beginning of the k-means clustering, the initial cluster centers are often selected randomly, which may lead to unstable clustering results. Therefore, we used the maximum and minimum initial point optimization algorithm (39) to determine initial cluster centers, which helped to make the clustering results more stable. First, the data point with 0 scores for all questions (i.e., no depression symptoms at all) was chosen as the first cluster center, and the data with the largest distance from the first clustering center was selected as the second clustering center (i.e., severe depression). Then, the nearest distance of each point to the known center was recorded as the “minimum distance”, and the data with the max “minimum distance” was selected as the next clustering center. This step was iterated until the number of centers reached k.

After the initial cluster centers were determined, the Euclidean distance between each sample and each cluster center was calculated. Every sample was allocated to the nearest center. Once all points were allocated, the center of each cluster was recalculated. This process was repeated until each center no longer changed or the number of times of repeats was up to the limit of 100. Finally, every individual was assigned the label of the cluster whose center was nearest to his/her answers to all items of the scale.

### Assessment of the Clustering-Based Classification

First, to test the robustness of the clustering-based classification method, 70% of the total sample (n = 5,387) were randomly selected to conduct the k-means clustering. Based on the clustering results, a linear discriminant analysis (LDA, singular value decomposition with convergence threshold set at 0.0001) classifier (40) was constructed on the 70% of the sample and the LDA classifier was then used to assign the remaining 30% of the sample (n = 2308) into the clusters. We then calculated the adjusted Rand index [ARI, (41)] between original clustering solution and the new clustering solution based on 70% resampling and 30% LDA results. The above random re-sampling procedure was repeated 10,000 times, and the 10,000 adjusted Rand indices were averaged.

Second, we examined the correspondence between the clustering-based classification and the norm-based classification to see whether the automatic classification method would generate results that resemble those from the traditional norm-based method, at least to some extent. Considering that various norms existed based on previous studies (see Introduction), we included both the original norms as well as adjusted norms. To cover the most likely scenarios, we established two new criteria with new norms: Criterion 1 shifted the cutoff points to one point lower for each level (25, 42), Criterion 2 shifted the cutoff points to one point higher for each level (25, 42). Kappa coefficients were calculated to assess the strength of the correspondence.

Third, we used 10-fold cross-validation to directly compare the model performance of the clustering-based vs. the various norm-based methods. Accuracy, AUC, and sensitivity were calculated to index model performance. As in the robustness test, an LDA classifier (singular value decomposition with convergence threshold set at 0.0001) was used to classify four levels of depression. The 13 BDI items were used as 13 features and the four levels of depression derived from each of the clustering- and norm-based methods were used as the labels. Because the classes (clusters) had different numbers of individuals (i.e., the class-imbalance issue), additional analyses were conducted after we under-sampled to keep the numbers of each depression level to the same as the smallest of the four classes.

Finally, six demographic variables (**Table 1**) and four depression-related scales (SAS, PSS, ISI, and ASLES) were used to assess the clustering method as compared to the norm-based method. Chi-squared (43) test was used to calculate the associations between demographic variables and depression level using SPSS 20.0 (SPSS Inc., Chicago, IL, USA). We conducted ANOVA to assess the associations between depression classification and related constructs. Eta-squared was used to index the strength of associations.

**Table 1:** Demographic variables.

Demographic variables	Response options
Gender	Male; female
Academic performance	Excellent; good; medium; poor
Burden of school work	Very light; somewhat light; average; heavy; very heavy
Parents divorced	Yes; no
Family economic situation	Much better than average; better than average; average; poorer than average; much poorer than average
Only-child	Yes; no

## Results

### Classification Results Based on K-Means Clustering and Their Robustness

After 25 iterations, the k-means clustering was completed and the numbers of individuals in the four clusters were 4,042, 2,455, 671, and 527. The scores of the four cluster centers were 1.47, 7.84, 11.27, and 18.7, corresponding to four depression levels of None, Mild, Moderate, and Severe.

To assess the robustness of the above results, we randomly selected 70% of the total sample to conduct the k-means clustering and used LDA classifier to assign the remaining 30% of the sample into the clusters. The resampled results were compared to the original classifications based on the total sample. Results showed that the average ARI between the original clustering solution and the new clustering solution based on 70% resampling and 30% LDA results was 0.91, SD = 0.07.

### Correspondence With Norm-Based Classifications

The confusion matrix between the clustering-based and the original norm-based classifications is shown in **Table 2**. The Kappa coefficient was 0.683, indicating moderately high correspondence. The correspondence was generally high for three levels of depression: None, Mild, and Severe. For Moderate depression based on the norm, the clustering method showed a wide spread across Mild, Moderate, and Severe categories.

**Table 2:** The cross tabulation of individuals in each level of depression based on the clustering- and norm-based classifications.

	Norm	None	Mild	Moderate	Severe	Total
Clustering						
None		3958	84	0	0	4042
Mild		25	1265	1165	0	2455
Moderate		5	83	521	62	671
Severe		0	0	144	383	527
Total		3988	1432	1830	445	7695

As mentioned earlier, considering that the original norms used in the above analyses were based on the study of Beck et al. (13), we conducted additional analyses based on adjusted norms (scenarios that might have occurred if an actual study was done to re-norm BDI for this sample) by establishing two new criteria with new norms: Criterion 1 shifted the cutoff points to one point lower for each level (25, 42), Criterion 2 shifted the cutoff points to one point higher for each level (25, 42) (**Table 3**). The Kappa coefficients between the two classifications based on adjusted norms and the clustering-based classification were 0.532 and 0.603, respectively. Clearly simple 1-point adjustment either way did not improve the correspondence between the norm- and clustering-based classifications (c.f., with a Kappa of.683 for the original norms), although they still showed a moderate-to-high level of correspondence between clustering-based classification and norm-based classifications.

**Table 3:** The number of people (N) and the corresponding score range (S) in each level based on the four ways of classifications.

Criterion	None			Mild			Moderate			Severe		
	S	N	%	S	N	%	S	N	%	S	N	%
Clustering	0–6	4,042	52.2	4–15	2,455	31.9	3–18	671	8.7	13–39	527	6.8
Norm	0–4	3,988	51.8	5–7	1,432	18.6	8–15	1,830	23.8	16–39	445	5.8
Criterion 1	0–3	3,438	44.7	4–6	1,534	19.9	7–14	2,155	28.0	15–39	568	7.4
Criterion 2	0–5	4,514	58.7	6–8	906	11.8	9–16	1,930	25.1	17–39	345	4.5

S, score range; N, number of individuals for a given level of depression based on a given method of classification; %, percentage of the total sample.

### Model Performance

**Figure 1:**
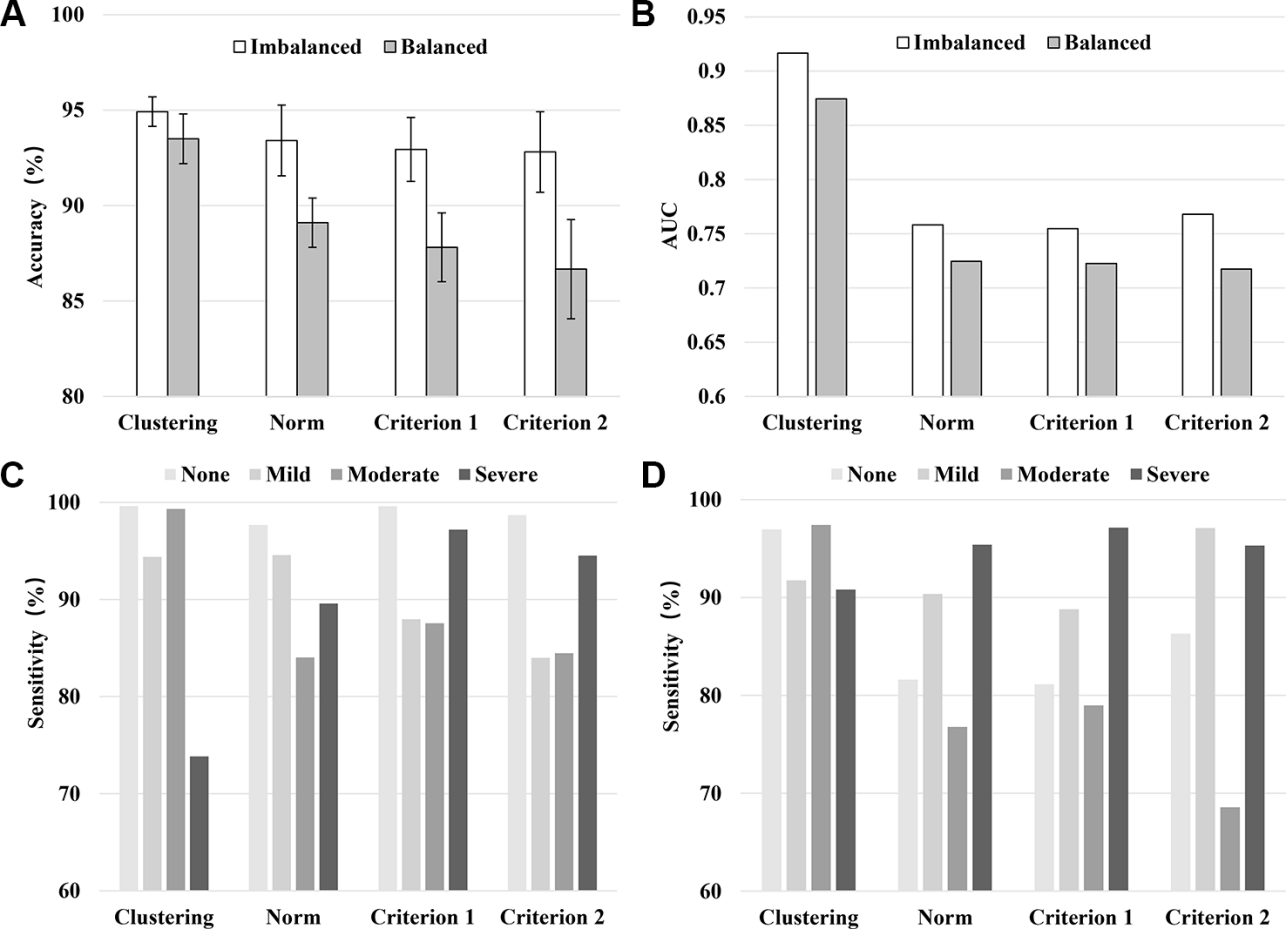
Model performance in terms of the averaged accuracy **(A)**, AUC **(B)**, and sensitivity (**C**: Imbalanced data, **D**: Balanced data) by classification method/criterion.

**Figure 2:**
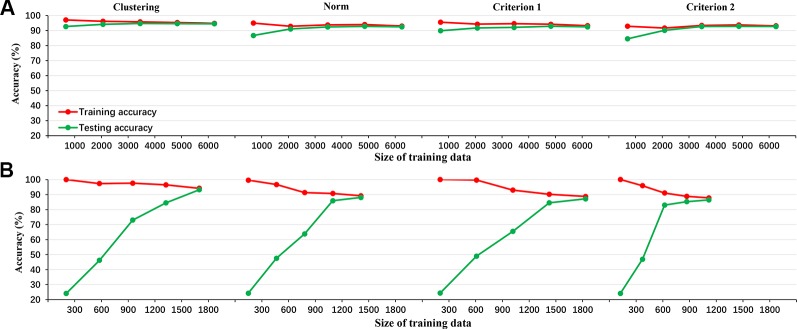
The learning curve of LDA classifier for the 4 criteria in the imbalanced data (**A**: clustering, norm, criterion 1, and criterion 2 from left to right) and balanced data (**B**: clustering, norm, criterion 1, and criterion 2 from left to right).

**Figure 1** shows the results of 10-fold cross-validation of the clustering-based vs. the various norm-based methods. Results are shown for both the original data (with unbalanced classes) and for the under-sampled data. It seems that the clustering-based classification performed well, with higher overall accuracy and AUC than the norm-based methods, for both the original unbalanced data and the under-sampled balanced data. In terms of sensitivity, the clustering method showed high sensitivity (> 90%) for all four levels of depression for the balanced data and for three levels of depression for the unbalanced data and at 73.83% for severe depression. The norm-methods showed varying levels of sensitivity by method/criterion and level of depression. To evaluate the generalization ability of the classification model (over-fitting or less-fitting), the learning curves of classifications are shown in **Figure 2** for the imbalanced and balanced data. Due to the smaller sample sizes for the balanced (under-sampled) data, the initial accuracies of the testing dataset were relatively low but increased steadily. Because the size of the smallest class varied by criterion, the sample size of the training data varied across the four criteria for the balanced data.

### Validity Assessment

**Table 4** shows the Chi-squared values between demographic variables and depression for different methods of classifications. The direction of associations was as follows: female > male, poor school performance > good school performance, heavy academic burden > light academic burden, parents divorced > parents not divorced, children with sibling(s) > only children, and poor family economic situation > good family economic situation. Given the large sample size, all associations were significant. In terms of the Chi-squared values, they were mostly similar across the methods. In addition, given their generally weak associations, demographic variables are not informative in assessing the validity of the classifications.

**Table 4:** The associations (Chi-squared) between demographic factors and depression levels based on different classification methods.

Demographic variables	Clustering	Norm	Criterion 1	Criterion 2
Gender	55.808	59.619	70.998	60.391
Academic performance	248.399	263.135	270.785	267.610
Burden of school work	377.312	411.159	349.587	386.253
Parents divorced	28.871	28.519	30.704	20.564
Family economic situation	167.901	161.236	157.392	152.535
Only-child	13.441	8.487	11.531	10.778

Given the sample size, all Chi-squared statistics were significant at the level of p < .001.

**Figure 3:**
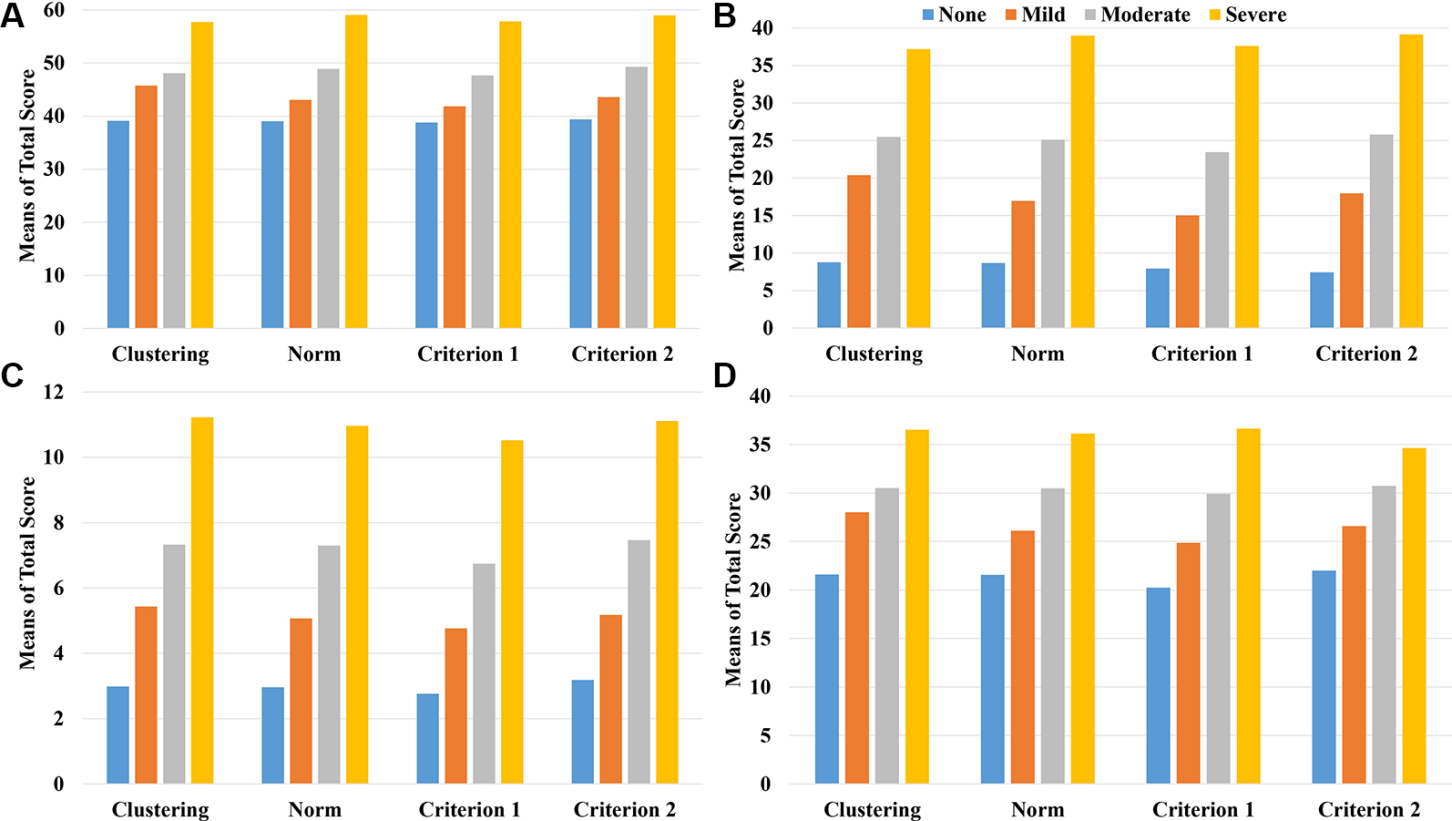
The mean scores of the related scales including SAS **(A)**, ASLEC **(B)**, ISI **(C)**, and PSS **(D)** by the level of depression and classification method/criterion.

**Figure 3** and **Table 5** show the associations between depression levels and scores of the other four scales (SAS, PSS, ISI, and ASLEC). Results showed strong associations (visualized in **Figure 3** and quantified as eta-squared in **Table 5**) regardless of classification methods. In other words, there appeared little differences in convergent construct validity (i.e., the strength of associations between depression levels and associated constructs) across the classification methods based on the ANOVA results.

**Table 5:** The Eta-squared values from ANOVA of correlates of depression by levels of depression.

	SAS	PSS	ISI	ASLEC
Clustering	0.30	0.27	0.21	0.29
Norm	0.34	0.30	0.24	0.31
Criterion 1	0.33	0.30	0.23	0.31
Criterion 2	0.33	0.28	0.22	0.30

## Discussion

In this study, we attempted an automatic approach to depression classification using k-means clustering, examined its robustness and correspondence with the traditional norm-based approach, and compared these approaches in their model performance and convergent construct validity (or associations with known correlates). Taken together all the information, the automatic approach appeared to perform well.

First, in order to solve the problem of outdated or inappropriate norms for classifying depression levels, we proposed an automatic approach using the k-means clustering method. This is an unsupervised learning method, which does not require previously labeled data. Compared to other clustering methods, the k-means clustering method is relatively easy to use (few parameters to adjust, low computational complexity) and allows for the users to set a fixed number of theoretically meaningful and practically useful clusters. Furthermore, compared to traditional norm-based methods, the k-means clustering method uses information from all individual items rather than a summary score. In other words, the traditional norm-based method has a fixed boundary and treats all items as having equal importance, whereas the clustering method is based on the distance from the sample to the center of clusters based on the multi-dimensional space of all items and has no single score boundary. Because this automatic method is data-driven and requires no prior labels, it can be flexibly adapted to any new dataset from any groups. Results from such classifications should be sensitive to geographic, cultural, historical variations in the distribution of depression levels (44, 45). The results of the current study provide the first evidence that such an automatic approach seems to perform well.

Second, the k-means clustering method yielded high robustness with an ARI of 0.91 and an SD of 0.07 across 10,000 times of re-sampling. In other words, this clustering method is resilient to sampling variations and yields stable clusters (46).

Third, the resulting clusters make intuitive sense by having a high level of correspondence with the traditional norm-based method. Specifically, the Kappa coefficient of the clustering- and the norm-based methods was 0.683, indicating a substantial agreement (47). As shown in **Table 2**, the two methods yielded more consistent results on the two extreme clusters of non-depression and severe depression than the other two clusters (mild and moderate depression). It seems that the max-min initial point optimization algorithm is suitable for identifying severe depression and non-depression, perhaps because the individuals with 0 points and highest points can be clearly defined and can adequately guide the clustering process.

Fourth, compared to the norm-based methods, the clustering-based method showed high accuracy and AUC as well as sensitivity for each depression level, indicating that the clustering results were reliable. In addition, **Figure 2** illustrated the accuracy metrics for the training and testing processes. Obviously, the classifier had a good generalization ability and the over-fitting or less-fitting did not occur in this case as (1) training accuracy and testing accuracy were high at the same time, and (2) there was no exist significant difference between training accuracy and testing accuracy in all iterations (48).

Finally, in further support of the validity of the clustering method, both classification methods yielded similar results in terms of known correlates (demographic variables and depression-related constructs). Higher depression level was associated with more stressful life events (30), higher perceived stress (31), higher anxiety (32), and poorer quality of sleep (33), as well as being female [e.g., (49)], coming from a divorced family [e.g., (50)], coming from poorer family economic situation (e.g., (51)], experiencing greater school burden [e.g., (52)], showing lower academic performance [e.g., (53)], and having sibling(s) vis-à-vis being single children [e.g., (54)].

## Conclusion

This study used a machine learning method to demonstrate a new automatic approach to determining classifications of depression levels that are specific to a given population. This approach overcomes the limitations of the out-of-date or inappropriate norms used in the traditional norm-based method.

## Data Availability Statement

All datasets generated for this study are included in the article/supplementary material.

## Ethics Statement

This study was approved by the Institutional Review Board of the State Key Laboratory of Cognitive Neuroscience and Learning at Beijing Normal University. Written informed consent to participate in this study was provided by the participants' legal guardian/next of kin.

## Author Contributions

CC, XZ, LY, and ZY conceived and designed this study. LY and XZ contributed data and analytical tools. ZY and HL conducted the coding and statistical analysis. ZY, CC, and XZ wrote the paper. All authors have read and approved the final manuscript. 

## Funding

This study was funded by the Funds for National Natural Science Foundation of China (grant number 61871040), the Key Program of National Natural Science Foundation of China (grant number 61731003), the National Key R&D Program of China (2018YFB1005100), and the Engineering Research Center of Intelligent Technology and Educational Application, Ministry of Education of China.

## Conflict of Interest

The authors declare that the research was conducted in the absence of any commercial or financial relationships that could be construed as a potential conflict of interest.
